# A perspective review on the role of engine sound in speed perception and control: state of the art and methodological suggestions

**DOI:** 10.3389/fpsyg.2024.1391271

**Published:** 2024-09-18

**Authors:** Valter Prpic, Elena Gherri, Luisa Lugli

**Affiliations:** Department of Philosophy, University of Bologna, Bologna, Italy

**Keywords:** speed perception, speed control, in-car sound, artificial engine sound, electric vehicle

## Abstract

In this review we focus on the role of in-car sound, specifically the artificial engine sounds, on drivers’ speed perception and control, a topic that has received little attention so far. Previous studies indicate that removing or reducing engine sound leads drivers to underestimate speed and, consequently, to drive faster. Furthermore, evidence suggests that specific sound frequencies could play a role in this process, highlighting the importance of in-car sound features. First, we show that the amount of research in the field is scarce and rather outdated, and that this is largely due to the fact that industrial research is subject to very few publications. Then, we examine benefits and limitations of different research paradigms used and we propose a protocol to investigate systematically the phenomenon. In particular, we argue for the benefits of a wider use of psychophysical methods in speed perception, a field that has been typically explored by means of driving simulation. Finally, we highlight some methodological and statistical limitations that might impact the interpretation of the evidence considered. Our methodological considerations could be particularly useful for researchers aiming to investigate the impact of sound on speed perception and control, as well as for those involved in the design of in-car sounds. These are particularly relevant for the design of electric vehicles, which represent a challenge but also the ideal testing ground to advance the knowledge in the field.

## 1 Introduction

Automotive industry is quickly approaching the electric vehicle (EV) era, as internal combustion cars will soon become obsolete. The European Union, for instance, plans to prohibit the sale of new petrol and diesel-powered vehicles by 2035, imposing a strict deadline for the industry to adapt to this novel mode of car production. Besides obvious advantages of EV, such as the reduction of air pollution in city centers, this new technology is posing considerable challenges for the society, including changes in both pedestrians and drivers’ behavior. However, while research on the sound of EVs has mostly focused on pedestrian safety (e.g., Karaaslan et al., 2018; Faas and Baumann, 2021; Oberfeld et al., 2022) only a minority of studies investigated the impact of sound on driving behavior.

From a driver’s perspective, the most salient change related to in-cab perception is the absence of the engine sound. As EV engines are extremely silent, most car manufacturers will apply artificial engine sounds (AES) which can provide auditory feedback to the driver (Min et al., 2018). Indeed, it is noteworthy that previous studies demonstrated that in-car auditory feedback is important to inform the driver about the car speed (Hellier et al., 2011; Merat and Jamson, 2011; Denjean et al., 2012). This has considerable implications for road safety as reduced speed perception due to the attenuation of auditory feedback is causing drivers to drive faster (Horswill and McKenna, 1999; Horswill and Coster, 2002), which is an important predictor of car accidents (Wasielewski, 1984; French et al., 1993; West et al., 1993). In this article, we review studies that investigated the role of sound in speed perception and control. We critically evaluate the methods used in the field, focusing specifically on the limitations of driving simulation studies which represent the method of choice adopted in most studies. Finally, we suggest methodological improvements and future research directions.

## 2 State of the art

Although drivers can use the speedometer to gauge real-time speed information about their vehicles, they seldom check it (Mourant and Rockwell, 1972) and tend to overestimate its frequency of use (Denton, 1969). Therefore, the drivers’ perception of the vehicle speed is often based on sensory information gained through the different senses, such as visual (e.g., optic flow), auditory, as well as vestibular, haptic and kinaesthetic information (for a review see Kemeny and Panerai, 2003). Nowadays researchers widely agree that the perception of the world is largely based on multisensory integration and that one sensory modality can significantly alter the perception of another modality. For example, auditory information can influence the perception of the visual properties of a stimulus (fission illusion, e.g., Shams et al., 2005; stream/bounce illusion, Sekuler et al., 1997). Likewise, visual information can alter the properties of auditory stimuli as shown for speech perception (McGurk and McDonald, 1976) or sound localization (ventriloquist illusion, for review see Chen and Vroomen, 2013). These classic multisensory examples demonstrate how our percepts can be distorted by the simultaneous presence of *conflicting* multisensory information. However, in real-life situations, the presence of *congruent* sensory information from different modalities typically leads to coherent, valid, and robust perception of our surroundings. For example, recent studies showed that visual information relative to motion can be biased by auditory information, suggesting that these two modalities are closely related (Hidaka et al., 2009, 2011). Although driving performance is largely based on vision (for a review see Owsley and McGwin, 2010), several auditory cues can influence the driver’s speed perception. Indeed, the rolling sound of the tyres, wind turbulence and the sound of the engine are all related to car speed. In modern cars and, particularly, in EVs some auditory cues are attenuated or completely absent (e.g., engine sound), thus potentially altering speed perception.

Although few studies focused on the role of auditory feedback on driving behavior and speed perception, converging evidence shows that the attenuation or absence of the engine sound leads drivers to underestimate speed (Evans, 1970; Horswill and McKenna, 1999). Consequently, they tend to drive faster (Horswill and McKenna, 1999; Hellier et al., 2011) and to show poorer speed control (McLane and Wierwille, 1975; Merat and Jamson, 2011; Denjean et al., 2012). For example, a driving simulation study (Hellier et al., 2011) showed that the absence/low levels of engine noise induced participants to drive faster and to commit more driving violations. Interestingly, this result was found without occluding the speedometer, thus suggesting that its presence does not influence speed choice (Hellier et al., 2011). Furthermore, in this study participants reported decreased levels of driving comfort in the low engine noise condition, suggesting that the current trend of in-car noise reduction in the automotive industry can potentially compromise the driving experience. This is an interesting finding since previous studies found an opposite pattern of results, with loudness negatively related to comfort (trade off hypothesis between pleasantness and power; Park et al., 2019). However, it is noteworthy that most of the research on in-car sound comfort and pleasantness has been conducted in listening rooms, which cannot recreate the experience of driving and may lead to different results (see also Melman et al., 2021).

Consistent with the literature in the field, another simulation study (Merat and Jamson, 2011) revealed that drivers struggled to maintain speed in the absence of a speedometer when driving at faster speeds (70 mph vs. 30 mph). Moreover, the drivers’ ability to maintain the target speed was further impaired by the absence of vehicle noise, again causing them to drive faster (Merat and Jamson, 2011). Another driving simulation study (Denjean et al., 2012) revealed that the underestimation of speed in the absence of auditory feedback was greater in daytime than in night-time light conditions. The authors suggested that drivers tend to focus more on the lateral visual stream at night-time, which is a better visual cue for estimating driving speed, because they do not see a long distance ahead under nightlight. Consequently, auditory information was less important under nightlight conditions. Furthermore, drivers tend to drive more slowly during the night due to degraded visual conditions and this can in turn result in improved speed control. This is consistent with previous studies that revealed slower speed under nightlight conditions in driving simulation experiments (for a review see Kemeny and Panerai, 2003).

Another study further investigated the role of sound in speed estimation by analyzing the role of different sound frequencies (Wang and Wang, 2012). This study confirmed previous findings that speed underestimation is particularly evident at high speeds and when the auditory feedback is absent. Indeed, Wang and Wang (2012) showed serious underestimation of speed at 120 km/h, further extending the speed range used by Evans (1970) who showed speed underestimation at 100 km/h.^1^ Moreover, Wang and Wang (2012) revealed that removing frequencies below 600 Hz improves speed estimation particularly at high speeds. This is because low frequencies (below 600 Hz) tend to decrease as speed increases, so removing this frequency band creates the illusion that the car is moving faster which compensates for the typical speed underestimation found at high speed. This study provides helpful insights for soundproofing design as it suggests that there is no need to eliminate all in-car sounds but only unnecessary noises. This would help to both maintain a quiet cabin and to preserve speed estimation, increasing driving safety. Another recent study investigated the use of sonification to enhance the perception of car dynamics (Denjean et al., 2021). Results showed the role of pitch in modifying the perceived acceleration/deceleration and the relative speed production (Denjean et al., 2021). However, although clear conclusions cannot be drawn from this study on speed perception, the approach of investigating the contribution of different sound parameters (e.g., pitch height) is extremely promising as it will help to predict the driving behavior under the extremely diverse soundscapes developed by different car manufacturers.

Surprisingly, while EVs are rapidly populating roads all over the world, most of the literature about speed perception is rather old and only limited research has been published in recent years. This is likely due to the fact that sound design in EVs is highly industrial and, thus, subject to very few publications. Given the paucity of academic publications in the field we have included in this review also contributions published in specialized conferences. A recent study by Melman et al. (2021) investigated the role of an artificial engine sound on speed perception and control (AES; similar to the ones adopted by electric vehicles) in a driving simulator. In particular, this study investigated whether an AES mimicking a sport car with enhanced engine power would influence speed control. This study showed improved speed control (lower standard deviations) in the AES condition compared to the baseline (sound of a normal city car) but only while driving through curves. This was probably because the engine sound was particularly enhanced in the AES condition, thus providing particularly efficient auditory feedback. Interestingly, since it is harder to look at the speedometer while driving through curves, it is likely that the auditory feedback gained further importance in those parts of the circuit. It is therefore possible that sound is particularly important under conditions in which the speedometer is hardly accessible (e.g., see Denjean et al., 2012 and Merat and Jamson, 2011 for similar results with the speedometer occluded). In the study of Melman et al. (2021), the speedometer was visible so it might be that auditory feedback was less effective in supporting speed control, except for the curvy sections where higher visual demands prevented participants to glance at the speedometer. Finally, in the study by Melman et al. (2021), participants reported increased perceived sportiness (but not increased comfort) in the AES condition without altering average driving speed.

Another recent study investigating the role of sound in speed control by using a driving simulator was carried out by Sukegawa et al. (2019). They compared participants’ behavior in three sound conditions: internal combustion (engine sound and background noise), electric vehicle (background sound only) and silence (no sound). Sukegawa et al. (2019) found improved speed control during acceleration and cruising when the engine sound was present. While this study confirmed previous findings regarding the role of engine sound in speed control, several questions on how electric vehicle sounds influence driving behavior remain open. The EV condition of Sukegawa et al. employed no engine sound providing only background noise to the participants. This condition is quite unrealistic as EVs will certainly employ some kind of artificial sound and currently the only two studies investigating the role of AES in speed control are those by Denjean et al. (2021) and Melman et al. (2021) described above. However, while in the latter the AES consisted of a simulation of an internal combustion engine of a sport car, in the former the sonified sound consisted of an auditory illusion (Sheppard-Risset glissando illusion) that used changes in pitch to mimic the vehicle acceleration/deceleration. These two manipulations represent very different sound solutions, neither of which is likely to be adopted by car manufacturers. Indeed, different brands are creating their own signature sounds, often by adopting a mixture of synthesized engine sounds and music (see Viola, 2021, for an overview of different EV brand sounds, as well as various psychological aspects that affects the adoption of EVs). The interesting point to make here is that current research on speed perception does not allow to predict how the highly diverse range of auditory feedback offered by different brands will influence drivers’ behavior. Despite the fact that the transition to EVs is quickly approaching, research in this field is still in its infancy.

Most of the studies reviewed used driving simulation (see Table 1 for details), which has the advantage of being a realistic and direct measure of driving behavior. However, from a methodological point of view, using driving simulators to investigate speed perception has the disadvantage that the judgment of speed perception in this context is largely dependent on absolute speed. In driving simulators studies, participants are typically required to drive at a defined absolute speed (e.g., 30 or 70 mph in Merat and Jamson, 2011) while their speedometer is occluded. This task is characterized by large interindividual variability (Poulton, 2023).

**Table 1:** Description of reviewed studies specifically investigating the role of sound on speed perception and control by using driving simulation.

Authors and year of publication	Sample size (% male) Age range, mean (sd)	Driving license Driving expertise (mean/SD years) Profession	Study design/conditions Repeated trials	Sound stimuli Sound type	Simulator type Screen type Sound device	Task Scenario Traffic	Dependent variables Outcome and main results
Merat and Jamson (2011)	12 (83%) 19–30, 23.58 (2.3)	YES 5.63 (SD = 2.3) N/A	Within subject design (Speedometer + sound; No speedo + sound; No speedo, no sound) 1	Engine + tires + wind Recorded	Dynamic N/A N/A	Accelerate, maintain speed (30–70 mph), decelerate 3-lane section of a motorway NO	Driving speed (mph) Faster driving and larger speed variability from target speed with no sound
Hellier et al. (2011)	48 (56%) 18–35, 23.5 male (N/A), 25.1 female (N/A)	N/A 5.25 (SD = N/A) Students	Between subject design (4 conditions, no engine noise +3 levels of engine noise) 1	No engine noise (ambient sound on) + 3 levels of engine noise (65, 75, 85 dB) Simulated	Static computer video-game 120×170 cm projected image Altec Lansing Speakers (model 221)	Drive as you would in reality, observing normal traffic laws Mixed scenario N/A	Driving speed (mph) + scale measures of comfort, loudness and realism Faster driving for no engine noise and low noise. Low noise was associated with reduced comfort.
Denjean et al. (2012)	24 (92%) 18–54 N/A	YES N/A N/A	Within subject design3 sound conditions 2 visual conditions (day/night) 1	Engine car sound (engine + tires + wind); Electric car sound (tires + wind); No sound N/A	Fixed-based Full field view N/A	Accelerate, maintain speed (70–90 km/h) N/A NO	Speed deviation between actual and target speed (km/h) Larger speed underestimation and fast driving with day light and no sound. Engine sound help maintaining speed.
Melman et al. (2021)	32 (81%) 19–35, 23.4 (3.1)	N/A N/A (days per week and mile per year are reported) N/A	Within subject design 5 sound conditions 4 acceleration trials	Engine car sounds (Baseline, Artificial Engine Sound AES, Modified Throttle Mapping MTM, AES + MTM, Sport sound) N/A	Fixed-based 90-degree field of view Headphones	Accelerate, maintain speed (of their choice) on straight line and curves Single-lane road with trees, buildings, landscapes, and guardrails NO	Driving speed (km/h), acceleration (m/s^2^), throttle measures (%) + scale measures of driving experience Sound enhancement increased speed control (lower speed variability in curves) but not mean speed
Sukegawa et al. (2019)	24 (100%) ~20–29 N/A	YES (only 20 participants) N/A N/A	Within subject design 3 sound conditions 5	Engine + tire + wind; Tire + wind; No sound Recorded	Static computer simulation N/A Headphone	Maintain speed (40–80 km/h), accelerate, decelerate Simple white dashed line on the road, no buildings NO	Speed deviation between actual and target speed (km/h) Better speed control with engine noise at acceleration and speed maintaining.

We sorted columns by: Authors and year of publication; Sample size (percentage of male), Age range, mean (standard deviation); Driving license, Driving expertise (mean and standard deviation in term of years of driving), Profession; Study design/conditions, Number of repeated trials; Sound stimuli, Sound type; Simulator type, Screen type, Sound device; Task, Scenario, Traffic; Dependent variables, Outcome and main results. N/A, Not Available.

An alternative technique to investigate speed judgment is to videotape the driver’s visual field through the windscreen and ask participants to estimate speed by making relative judgments. For example, Horswill and Plooy (2008) investigated the role of in-car auditory feedback in speed perception by using a classic psychophysical method based on relative speed judgments, which is unaffected by the measurement errors typically associated with absolute speed judgments. This study used the method of constant stimuli in which participants were presented with pairs of video-recorded driving scenes and were required to perform a 2AFCT (two-alternative forced choice task, i.e., judge whether the second scene appears faster or slower than the first). The experimental manipulation consisted in the presentation of the video scenes coupled with either a real world in-car noise level or a reduced volume level of 5 dB. This study demonstrated that in-car noise attenuation leads to speed underestimation, in line with existing literature, as suggested by the fact that participants judged the speed to be significantly slower in the attenuated volume condition. It is noteworthy that the psychophysical methods of constant stimuli led to a very low interindividual variability, unlike studies based on driving simulation. Therefore, this method seems to be a particularly consistent measure of speed perception. Although the work of Horswill and Plooy (2008) successfully overcame some of the limitations of previous studies (e.g., Horswill and McKenna, 1999), the use of psychophysical methods has not been adequately considered by other researchers in the field which tends to rely on absolute speed judgments derived from diving simulations studies (e.g., Denjean et al., 2012). However, while psychophysical methods represent a particularly solid measure of perception, they suffer from a lack of realism, which is instead a key feature of driving simulation. It is conceivable that speed judgments are differentially affected by the active or passive role of the observer in the speed perception task (passively making judgments vs. actively driving the car while trying to maintain a constant speed). However, there is no evidence yet supporting this possibility. Instead, a field study by Schütz et al. (2015) clearly showed that the misestimation of speed was highly correlated between drivers and passengers. This evidence suggests that the behavior of the observers should not differ from that of drivers and justifies the use of video recordings in the speed judgment task.

Besides driving simulation and video-based techniques, field studies have also been employed to investigate speed perception (for example see Evans, 1970). This method is clearly the most realistic as participants are asked to estimate speed in a real driving scenario but from a methodological point of view this is also the most difficult set up to control. While all three methods (i.e., driving simulation, video-based techniques and field studies) have clearly their pros and cons, driving simulation is a good compromise between realism and rigor. However, it should be noted that driving simulation only provides an ‘illusion’ of driving (Hancock, 2009). Indeed, stimuli in real driving environment are of greater complexity (see Kemeny and Panerai, 2003) whereas risk perception is highly reduced in driving simulation, leading to different driving behaviors. Although a recent validation study (Hussain et al., 2019) showed that speed perception in driving simulation can lead to similar results as field studies (see also Panerai et al., 2001), there are other evidence in the literature that shows discrepancies between the two methods. For example, while Denjean et al. (2012) found that participants underestimated speed to a larger extent during daylight (vs. nightlight) conditions in a driving simulation experiment, Schütz et al. (2015) showed that speed estimation was particularly robust under lighting conditions in their field study. This example highlights the importance of using different methods to better understand the mechanisms involved in driving behavior. Relative speed judgments based on video recordings of drive scenes are likely to be the most rigorous methods to investigate speed perception (as shown by consistency and low variability in participants responses), whereas field experiments are better suited to understand complex behavior in real life. Conversely, the driving simulation method represents a mid-ground between rigor and realism, giving a balanced but rough approximation of the perceptual, motor and cognitive mechanisms involved in driving.

## 3 Methodological limitations

In this section we highlight some methodological limitations that are present in the literature and provide suggestions for future works.

The first point we want to discuss is that, in our opinion, the role of sound in speed perception deserves to be investigated in a more systematic way. Indeed, most studies we reviewed omitted details about the actual sounds present in the vehicle or simulated environment. For example, it is often unclear what is the specific contribution of engine, tire and wind noise in speed perception and control studies. More specifically, it is particularly important to assess how this contribution (both in terms of loudness and frequency spectra) changes at different speed levels (e.g., Ogle et al., 1996). However, we believe that engine sound is particularly important for the driver since it is directly related to vehicle speed and it represents a more steady and reliable predictor compared to the other two auditory cues. In real life, tire noise largely depends on the condition of the asphalt (e.g., dry/wet, texture, dirt, imperfections) as well as aerodynamic noise depends on weather conditions (e.g., humidity, rain, wind). The importance of engine noise is supported by two studies (Denjean et al., 2012; Sukegawa et al., 2019) that aimed to disentangle the specific contribution of engine noise from those of tire and wind noise. Indeed, the results of both studies suggest that the specific contribution of engine noise is important for speed control and maintenance. Nonetheless, considering the limited number of studies and the methodological issue we highlighted in this review, future studies should more systematically investigate the contribution of each auditory cue during different speed and environment conditions. We believe that EVs offer an ideal testing ground to disentangle the contribution of the engine sound from that of the tire and wind noise. Indeed, the absence of the engine sound allows to directly compare speed perception and control under different sound conditions (no engine sound, AES, simulated ICE sound) even in field studies, a situation that was inconceivable with ICE cars. Furthermore, only a few studies (Wang and Wang, 2012; Denjean et al., 2021) assessed which specific sound frequencies provide the auditory feedback that increases the drivers’ ability to control speed. This is of fundamental importance as sounds are not unitary phenomena, but multifaceted events characterized by several features (e.g., pitch height, brightness, temporal dynamics, loudness, timbre, sound location). Future studies should identify which of these sound features can be useful to the driver and how these could be preserved (or even enhanced) while reducing the needless noise at a minimum.

A second issue we identified is that, while several studies have shown an important contribution of vibrotactile and vestibular cues to driving behavior (for a review see Kemeny and Panerai, 2003), this information has not been adequately considered and/or reported when assessing speed perception. In particular, the driving simulation studies reviewed in the current work are a mix of evidence gained with very different simulation set-ups. Some studies were conducted with tools that allowed to simulate the motion dynamic of the vehicles (Merat and Jamson, 2011), whereas many others were carried out with fixed-based simulators (or even with much simpler computer video-game simulation; see details in Table 1) which did not provide any type of vestibular feedback. Therefore, the various levels of vestibular feedback present in the simulation set-ups represent a potential confound that should be taken into account when comparing and interpreting evidence from different studies. Indeed, contrasting evidence was found in steering research using fixed based vs. dynamic driving simulators. For example, Wallis et al. (2002) showed that participants failed to properly execute a lane change in absence of vision when using a fixed base driving simulator. However, a follow up study (Macuga et al., 2007) showed that accurate steering is possible without visual feedback when vestibular and somatosensory cues are provided in a dynamic simulator. Although steering research suggests that vestibular feedback plays an important role only when visual information is not available (Wilkie and Wann, 2005), the influence of vestibular cues on speed perception is largely unknown and poorly considered when interpreting the results of driving simulation studies. Future studies should systematically compare results from fixed based and dynamic driving simulators to investigate whether vestibular and vibrotactile information can generate interactive or additive effects with sound cues. Finally, there are other vibrotactile cues that are available while driving which have been scarcely considered by the literature in the field. For example, in car vibrations progressively intensify with car speed but, differently from the engine sound, they cannot be masked by competing stimuli (e.g., background music, talking to other passengers or at the phone). Thus, vibrotactile stimuli can be considered a pervasive and potentially relevant cue for controlling car speed. Future studies should assess the contribution of vestibular and vibrotactile feedback in vehicle speed perception and investigate their role, both in isolation and in a multisensory context. We also encourage researchers to accurately report all the relevant details about the driving simulators used in their studies and to consider these characteristics when interpreting the results (e.g., acknowledging the potential limitations of the equipment/feedback).

The third issue identified in the studies reviewed above is of statistical nature. Based on the information reported in those studies, it is not possible to establish whether existing studies are underpowered. This is because the studies reported in Table 1 do not report *a-priori* power analyses nor the effect size in their analysis, making the strength of the reported effects difficult to evaluate. This is a serious issue which could undermine the reliability of these findings and could explain—at least in part—some contradictory or null results reported in the literature. Therefore, future studies should include *a priori* power analyses to justify the sample size considered. Nowadays, the topic of statistical power is considered crucial and is closely scrutinized in psychological sciences, to the point that most psychology journals explicitly require to justify the sample size and include an *a priori* power analysis during the submission process. This good practice should be extended also to the fields of ergonomics and human factors because research in these areas inform industrial design which has a clear impact on human life. Furthermore, if an adequate sample size is necessary to consider variability across participants (i.e., differences between participants), similarly an adequate number of trials is necessary to consider trial-to-trial variability (i.e., temporary fluctuations in participant’s state, such as attention). Together with the effect size of a given phenomenon, both these elements determine the statistical power of a test. A recent work by Miller (2023) suggests that it is more likely to maximize power in reaction time studies by testing a large number of participants with fewer trials than a smaller sample with a larger number of trials per participant, although this might depend on the features of the phenomenon under investigation. Indeed, some effects vary considerably across participants while others remain more stable between individuals but mainly fluctuate across trials. Our opinion is that both sample size and number of trials should be adequately considered in driving research, as both variability across trials and across participants is likely to play a relevant role in driving (see also Brysbaert and Stevens, 2018 for a power analysis tutorial with mixed effects models).

Furthermore, many studies in the field omit important methodological details. For example, most studies include a poor description of the participants’ demographics, often lacking the information needed to ascertain whether participants were naïve or professionals working in the car manufacturing industry. The absence of information on the participants’ background and on their driving proficiency in both real and virtual worlds clearly poses issues both for the replication of the findings and for their generalization. Similarly, the representativeness of the sample is also quite problematic in the field. Indeed, in several studies participants are mostly young males (Merat and Jamson, 2011; Denjean et al., 2012) and as a consequence the findings are poorly generalizable to other sections of the population (e.g., different gender groups, age groups etc.). We believe that a broader use of video recording methods (which are easier to administer to a larger and more diverse sample) might be able to fill this gap and help to gain better understandings on the biases that affect speed perception.

Similarly, driving scenarios are poorly described in the majority of studies we reviewed, making the findings difficult to generalize and replicate. Because speed perception relies heavily on visual cues (optic flow), it is fundamental to evaluate how many of these cues are available in each driving scenario. For example, a participant in a driving scenario with limited optic information (such as a desert or an empty highway) might be more inclined to rely on sound cues, while auditory feedback might be less important in a scenario with informative optic cues. Poor description of the stimuli and high variability in driving scenarios between studies do not allow to critically evaluate the impact of this variable on speed perception and control. Similarly, specific task requirements and the instructions given to participants during the experiments could modulate the saliency of auditory/visual cues, thus influencing speed perception. All the details regarding the materials, the stimuli and the procedure should be carefully considered and reported to allow replicability and interpretation of the findings. Among the studies we reviewed Melman et al. (2021) made available their study materials and data-sets. This is an example of good practice which future studies could follow to ensure results replicability. Furthermore, the degree of realism of both stimuli and simulated environment is crucial to evaluate the extent to which research findings can be used to infer driver’s perception and behavior in real life. By examining the literature (see Table 1) it becomes clear that both increased methodological rigor and more detailed descriptions of the methods are needed to advance the research on in-car speed perception and control.

Reviewing the literature related to the role of engine sound on speed perception and control (see section 2 “State of the art”), we realized that most studies in the field were carried out with driving simulators, fewer were field studies, and only a handful of studies used video recording methods. While field studies are more costly compared to other methods, it was surprising to realize that video recordings were seldom used despite their practical advantages. Unlike field studies, studies with video recording methods are relatively easy and inexpensive to carry out and could contribute to widen and increase the number of studies on in-car speed perception. Importantly, these video recording studies combined with a psychophysics approach could improve the methodological rigor of the research in the field as well as the generalizability and representativeness of the findings reported. It is likely that video recording methods have been neglected so far because their use is limited to the study of the driver’s perception, while driving simulation methods are also suitable to investigate the driver’s behavior. However, initial evidence suggests that the (mis)estimation of speed is highly correlated between drivers and passengers (Schütz et al., 2015), warranting the systematic investigations of the driver’s perception. Due to its practical advantages, the use of psychophysical methods could be an important preliminary stage used to inform driving simulation and field studies, as we discuss further below.

## 4 A multi-method protocol

At present it is not clear which (if any) protocol the automotive industry is following to test their EV brand sounds. Recent studies investigating the design of in car soundscape mostly focused on pleasantness and comfort (Celiberti et al., 2022, 2024; Wang et al., 2023), rather than on the auditory cues that help the drivers to control speed. Therefore, it seems that the automotive industry is mainly focusing on the qualitative aspects of sound, largely neglecting its impact on driving behavior. However, given the importance of sounds for the safety of driving, we argue that a safety protocol should be employed when designing new EV sounds, similarly to other safety protocols already defined and adopted in car manufacturing.

We therefore suggest that a rigorous protocol should be followed to investigate the role of auditory sounds of EVs on driving behavior by using a combination of all the three methods (judgment of video recordings, driving simulation and field studies) at different stages. This protocol should also include an assessment of the perceived comfort of driver and passengers which is a key element in the design of EV sounds. To this aim, participants should be first tested in a listening room to compare the perceived comfort of the new EV sound with that of an ICE (Internal Combustion Engine) car of the same model. Then, a psychophysical method like the one used by Horswill and Plooy (2008) should help defining whether the new EV sound leads to an under/overestimation of car speed compared to the standard ICE car in a controlled environment. The use of psychophysical methods in the judgment of video recordings of drive speed is likely to be the most accurate technique to assess the existence of a bias in speed perception, although it lacks realism and cannot tell us much about driving behavior (i.e., if the perception bias turns into reduced speed control). To solve the issue of the lack of realism, one possibility could be to use virtual reality headsets in order to provide a more vivid experience when viewing the video recordings. In a following stage, a driving simulator study should investigate the perceived comfort and driving behavior (both in terms of speed control and driving violations) under both EV and ICE sound conditions. Finally, a field study should confirm previous evidence gathered under controlled conditions (e.g., labs and listening rooms) in a real driving scenario. We believe that only combining different experimental methods and tasks (e.g., speed judgment, speed maintenance) will allow to fully understand the complexity of the processes that drivers have to implement to control speed.

The three methodologies proposed are very different in terms of procedure complexity, required equipment and associated costs. Clearly, field studies are the most complex and require more resources, while video-recording methods are the most affordable. Indeed, video recording methods are easy and cheap to administer, so these are characterized by several advantages that should encourage its wider use: (1) they are suitable to test larger samples; (2) they do not require specific training or driving skills; (3) they are suitable to test specific groups (e.g., elderly people). Video recording methods positively impact the generalizability and representativeness of the findings from driving simulation or field studies, which are often conducted on small samples of expert drivers. Furthermore, the simplicity and low economic costs associated with video recording methods makes it ideal for running pilot studies in the first phases of the research. Conversely, complexity and high economic costs makes field studies ideal for the last phase of the protocol, that is when a phenomenon has already been systematically studied in the lab (e.g., it is easier to justify a costly field study if data from video recordings and driving simulations consistently show a bias in speed perception).

The protocol described above represents an ideal scenario to investigate biases in speed perception and control. We are aware that its full implementation can be not only costly but also lengthy. For this reason, the adoption of this protocol does not necessarily imply that each study/Lab should carry out all three phases (with the different methods) suggested. Because different research groups/Labs have dedicated research facilities and equipment as well as specific sets of expertise, the implementation of this protocol should be intended as a collaborative effort between research groups/Lab with different skill sets and research facilities. For example, psychology labs without specialized equipment to conduct driving research could offer a significant contribution to the field by using methods typically adopted to study human perception.

In the future, the soundscapes of EVs will be extremely diverse as different brands will offer different sound solutions (see Viola, 2021 for an overview of different sound solutions proposed by different car manufacturers). Therefore, the accurate consideration of different sound features will be of paramount importance to design an in-car sound that does not induce speed underestimation and that can thus be deemed as safe. Initially, this seems more suitable to be studied in listening rooms by using psychophysical methods, then further assessments should be done following the protocol described above. By using such a systematic approach, we believe that future cars will be able to provide accurate sensory feedback and at the same time to increase comfort for both driver and passengers. Although we focused on EVs, mainly due to their increasing popularity and the high variety of their in-cab soundscapes, most of the considerations we presented in this work, as well as the protocol we suggest, could be applied also to other types of vehicles. Finally, we believe that the manipulation of sound features in future cars could be used not only to guarantee an accurate speed perception but also to induce speed overestimation (leading drivers to slow down) at crucial moments when drivers usually show poor speed control.

## 5 Conclusion

This perspective review focuses on the role of engine sound in speed perception and control. Consistent evidence suggests that removing or reducing the engine sound leads drivers to underestimate speed and, thus, to drive faster. We point out that studies focusing on Artificial Engine Sounds (AES), which constitutes the in-car soundscape of electric vehicles (EV), are scarce and mostly focusing on pleasantness and comfort, rather than on speed perception. We also identify several methodological issues in the literature and we suggest a multi-stage protocol to investigate the phenomenon. Finally, we suggest that a wider use of psychophysical methods based on video recordings might be a practical solution to compensate some issues of driving simulation studies and to reliably assess potential biases in speed perception.

## Footnotes

^1^Interestingly Evans (1970) showed a reversed bias to overestimate speed below 90 km/h.
